# Correlation of iron deficiency indexes with Eradication and Recurrence of Helicobacter Pylori Infection in Children

**DOI:** 10.12669/pjms.40.4.7816

**Published:** 2024

**Authors:** Xiao Liu, Yuanda Zhang, Qingwei Dong3, Hongyu Deng, Jing Bi

**Affiliations:** 1 Xiao Liu, Department of Gastroenterology, Beijing Children’s Hospital Affiliated to Capital Medical University Baoding Hospital, Baoding, Hebei,071000, P.R. China. Key Laborary of Clinical, Research on Respiratory Digestive Disease, Hebei Baoding, 071000, China; 2 Yuanda Zhang, Department of Nutriology, Beijing Children’s Hospital Affiliated to Capital Medical University Baoding Hospital, Baoding, Hebei, 071000, China. Hebei Key Laboratory of Infectious Diseases Pathogenesis and Precise Diagnosis and Treatment, Hebei, 071000, P.R. China; 3 Qingwei Dong, Department of Gastroenterology Beijing Children’s Hospital Affiliated to Capital Medical University Baoding Hospital, Baoding, Hebei,071000, P.R. China. Key Laborary of Clinical, Research on Respiratory Digestive Disease, Hebei Baoding, 071000, China; 4 Hongyu Deng, Department of Clinical Laboratory, Affiliated Hospital of Hebei University, Baoding 071000, Hebei, China; 5 Jing Bi, Department of Infectious Diseases, Beijing Children’s Hospital Affiliated to Capital Medical University Baoding Hospital Baoding, Hebei, P.R. China; Hebei Key Laboratory of Infectious Diseases Pathogenesis and Precise Diagnosis and Treatment, Hebei, 071000, P.R. China

**Keywords:** Children, Helicobacter pylori, Iron deficiency, Eradication, Recurrence

## Abstract

**Objective:**

To explore the correlation of iron deficiency (ID) indexes with eradication and recurrence of Helicobacter pylori (Hp) infection in children.

**Methods:**

This is a clinical comparative study. One hundred and twenty-six children who were first diagnosed as Hp infection in Baoding Children’s Hospital (Hp infection group); and the control group included 200 children without Helicobacter Pylori infection (negative stool Hp antigen test and/or 13C-urea breath test) in local region at the same time from January 2020 to January 2022. Enrolled children were subjected to routine blood test, serum ferritin (SF), serum iron (SI) and total iron binding capacity (TIBC) detection. Meanwhile, children with Hp infection were given triple therapy for eradication and followed up for one year.

**Results:**

The levels of SI, SF and Hb in non-eradication group were lower than those in eradication group (P<0.05); while TIBC level in the former group was higher than that in the latter group (P<0.05). Furthermore, SF level in the recurrence group was lower than that in the non-recurrence group (P<0.05). While there was no significant difference in Hb, SI and TIBC levels between the recurrence group and the non-recurrence group (P>0.05).

**Conclusion:**

Low level of SF may be a risk factor for difficulty in eradication and recurrence after eradication in children with Hp infection. Meanwhile, low levels of Hb and SI are influential factors for difficulty in eradication in children with Hp infection.

## INTRODUCTION

Helicobacter pylori (Hp) is a gram-negative flagellated microorganism that is widely spread all over the world.^1^,^2^ Children are vulnerable to Hp infection^3^, and it has affected about one third of children around the world.^4^ The cause of Hp infection in children may be related to close contact with parents, which means they can get it from an infected adult^5^. Eradication of Hp in children mainly adopts “triple therapy” at present.^6^ The clinical application of triple therapy in Hp infection also triggers some problems gradually, such as high drug resistance rate and high recurrence rate.^7^,^8^ However, there are limited studies on the risk factors of recurrence of Hp infection in children. According to previous data, Hp infection has an intimate association with iron deficiency (ID) and iron deficiency anemia (IDA).^9^-^11^ Iron plays an essential role in the proliferation and oxidative metabolism of various tissues and cells. ID may produce a negative impact on children’s immune function.^12^ While so far, it is unclear whether ID will affect the eradication and recurrence of Hp-infected children. Accordingly, this study detected the ID indexes of children infected with Hp in this region, with one-year follow-up of the enrolled children simultaneously.

## METHODS

This is a clinical comparative study. The subjects of study were 126 children who were diagnosed as Hp infection at the initial visit of Baoding Children’s Hospital (Hp infection group); and the control group included 200 children who had no Hp infection (negative stool Hp antigen test and/or 13C-urea breath test) in local region at the same time from January 2020 to January 2022. In the Hp infection group, there were 73 boys and 53 girls, with an average age of (8.5±3.3) years old; while in the control group, there were 112 boys and 88 girls, with the average age of (8.9±3.2) years old. There was no statistically significant difference in the comparison of gender and age between the two groups (P>0.05).

### Ethical Approval

The study was approved by the Institutional Ethics Committee of Baoding Children’s Hospital (No.:2020-09; date: June 16, 2020), and Signed Informed written consent was obtained from the guardians of the children inducted in this study.

### Diagnostic criteria for Hp infection:

Positive Hp infection was defined according to the criteria of the presence of digestive system symptoms, as well as positive endoscopic pathological staining, rapid urease test and/or 13C-urea breath test^13^.

### Diagnostic criteria for ID and IDA^14^

ID: Serum ferritin (SF) <12 μg/L in children under five years old and <15 μg/L in children over 5 years old. IDA: Hemoglobin <110 g/L in children under five years of age, <115 g/L in children between 5~11 years old, and <120 g/L in children between 12~16 years old at the same time of meeting the diagnostic criteria for ID.

### Inclusion criteria:

- Children aged 4-16 years old.
- Children who agreed to take Hp test.

### Exclusion criteria:

- Children who took chalybeate or drugs that affected iron metabolism within six months.
- Children with serious chronic diseases.
- Children with false positive HP infection.
- Children who had recently suffered from sporadic acute infectious diseases.
- Children with anemia other than IDA; and (five) children with poor compliance who cannot complete follow-up on time.

### Therapeutic regimen

None of the children in Hp infection group had a history of allergy to amoxicillin. Children in Hp infection group were given clarithromycin at the dose of 10 mg/kg/time (twice a day), with the maximum dose of 0.5 g/time; amoxicillin at the dose of 25 mg/kg/time (twice a day), with the maximum dose of 0.5 g/time; and omeprazole at the dose of 0.3-0.5 mg/kg/time (twice a day), with the maximum dose of 20 mg/time. All drugs were administrated orally for two weeks. Children were reexamined with 13C-urea breath test after four weeks of drug withdrawal and followed up for one year. The 13C-urea breath test was performed again one year later.

### Sampling

An amount of two ml of venous blood were collected from each child on an empty stomach in the morning via the purple-cap and red-cap vacuum blood collection tubes.

### Routine blood test

Mindray BC-5300 automated hematology analyzer was used for determination in this study. Determination of serum ferritin (SF), serum iron (SI) and total iron binding capacity (TIBC): This study was carried out by Baoding Key Laboratory of Clinical Research on Children’s Respiratory and Digestive Diseases through chemiluminescence method using BECKMANCOULTER-Au5800 biochemical analyzer. The test reagents of SI and TIBC were provided by BECKMAN COULTER, and those of SF were purchased from Abbott. Gastroscopy: Olympus Q290 electronic gastroscope was used for gastroscopy in children with Hp infection in this study.

### Statistical analysis

This study utilized SPSS25.0 statistical software package for statistical analysis. The measurement data were represented by (*χ̅*;±*S*), and inter-group comparison used the analysis of variance. Statistically significant difference was determined when P<0.05.

## RESULTS

### Eradication

The 126 children with Hp infection enrolled in our study were reexamined with 13C-urea breath test after triple therapy, with 28 cases (22.2%) of positive and 98 cases (77.8%) of negative. Patient tested for positive were classified into non-eradication group, including 16 boys and 12 girls, with an average age of (8.5±3.7) years; while patients tested for negative were included into eradication group, including 57 boys and 41 girls, with an average age of (8.5±3.2) years. No statistical difference was observation in the comparison of gender and age between the two groups (P>0.05; Table-I).

**Table-I:** Data of eradication treatment.

Groups	Boys (n, %)	Age (years)
Non-eradication group	16 (58.2)	8.5±3.7
Eradication group	57 (57.1)	8.5±3.2
*χ^2^*/t	0.01	0.01
*P*	0.92	0.99

### Recurrence

The 98 children with Hp infection who were successfully eradicated were followed up for one year and then reexamined with 13C-urea breath test. The results showed that 20 cases (20.4%; 11 boys and nine girls) were positive (recurrence group), and 78 cases (79.6%; 46 boys and 32 girls) were negative (non-recurrent group), with an average age of (8.3±3.1) and (8.6±3.3) years, respectively. There was no significant difference in gender and age between recurrence group and non-recurrence group (P>0.05), as shown in Table-I to II.

**Table-II:** Data of Hp infection recurrence.

Groups	Boys (n, %)	Age (years)
Recurrence group	11 (55.0)	8.7±3.4
Non-recurrence group	46 (59.0)	9.0±3.5
*χ^2^*/t	0.10	0.36
*P*	0.75	0.72

### Prevalence of ID, IDA

In HP infection group, 23 patients (18.3%) had ID and nine patients (7.1%) had IDA; while in control group, 19 cases (9.5%) had ID and five cases (2.5%) had IDA. The incidence of ID and IDA in Hp infection group was higher than that in control group, with statistically significant difference (both P<0.05; Table-III).

**Table-III:** Comparison of the prevalence of ID and IDA between Hp infection group and control group.

Groups	Cases	IDA [n (%)]	ID [n (%)]
Hp infection group	126	9 (7.1)	23 (18.3)
Control group	200	5 (2.0)	19 (9.5)
*t*		4.05	5.28
*P*		0.04	0.02

The levels of SI, SF and Hb in non-eradication group were lower than those in eradication group, with statistically significant difference (P<0.05). Besides, there was statistically significant difference that TIBC level in non-eradication group was higher than that in eradication group (P<0.05) (Table-IV).

**Table-IV:** Comparison of SI, TIBC, SF and Hb levels in eradication group and non-eradication group.

Groups	Cases	SI (μmoL/L)	TIBC (μmoL/L)	SF (μg/L)	Hb (g/L)
Non-eradication group	28	11.4±2.7	76.9±13.3	20.3±8.6	117.6±8.4
Eradication group	98	12.7±2.2	70.2±10.3	25.5±10.5	124.0±7.7
*t*		2.66	2.46	2.36	3.79
*P*		0.01	0.02	0.02	0.00

SF level in the recurrence group was lower than that in the non-recurrence group, showing statistically significant difference (P<0.05). While there was no significant difference in Hb, SI and TIBC levels between the recurrence group and the non-recurrence group (P>0.05; Table-V).

**Table-V:** Comparison of SI, TIBC, SF and Hb levels in recurrence group and non-recurrence group.

Groups	Cases	SI (μmoL/L)	TIBC (μmoL/L)	SF (μg/L)	Hb (g/L)
Recurrence group	20	12.0±2.5	72.0±12.3	21.0±7.7	124.8±7.9
Non-recurrence group	78	12.9±2.1	69.8±9.7	26.6±10.9	123.8±7.7
*t*		1.65	0.86	2.16	0.47
*P*		0.10	0.39	0.03	0.64

## DISCUSSION

Hp infection is one of the most common chronic bacterial infections worldwide, with the involvement of >50% of the world’s population. Patients usually infect with Hp in childhood, and the infection will last for a lifetime if not treated. The eradication rate of “triple therapy” has been reported to be lower than 80% in many regions.^15^ The eradication rate of Hp infection in our study was 77.8% in patients after “triple therapy” in this region, which was consistent with the previous report. Recurrence of Hp infection may be associated with socio-economic and health conditions.^16^ Meanwhile, the one-year recurrence rate was 20% in children infected with Hp at the age of 5-9 years, and 8% in those at the age of over 10 years.^17^ A previous research by the members of our Research Group also revealed that the recurrence rate of Hp-infected children in the region was 18.8% after one year of successful eradication.^18^ In the present study, the one-year recurrence rate in this region was 20.4%, which was consistent with previous reports, which may be explained by the underdevelopment of the local economy.

As evidenced by multiple studies, Hp infection is related to ID and IDA, which can cause the decrease of SI and SF levels.^19^-^22^ Hp can cause blood loss in the digestive tract of patients, enhance the absorption of iron by bacteria, and reduce the absorption of iron by the body, thus leading to iron loss.^23^ This study compared ID indexes of children with Hp infection and those receiving physical examination in our hospital at the same time. The probability of ID and IDA in children with Hp infection was higher than that in children with physical examination, suggesting that Hp infection may increase the risk of ID and IDA in children. However, some other researchers argued that there was none significant correlation of Hp infection with ID and IDA^24^, and emphasized the role of socio-economic factors when analyzing the impact of Hp infection on the prevalence of ID in children.^25^ Unfortunately, this study failed to exclude socio-economic factors to further analyze whether Hp infection was related to ID and IDA.

IDA is a global public health problem in children, women and the elderly, which may have adverse effects on many diseases.^26^ The incidence of ID was 10.4% and 8.0%, while that of IDA as 2.9% and 2.8% in children aged 3-5 and 6-18 in China, respectively.^27^ Similar to previous reports, in our study, the incidence of ID and that of IDA in children aged 4-16 years old in this region were 9.5% and 2.0% respectively. ID and IDA can lead to impaired immune function in children^12^. Furthermore, the levels of SI, SF and Hb in children with Hp infection without eradication after triple therapy were lower than those of children who succeeded in eradication, suggesting that lower levels of Hb, SI and SF might be influential factors for the difficulty of Hp eradication. Simultaneously, children with Hp infection successfully eradicated by triple therapy were followed up for one year.

Consequently, SF level of children with Hp infection who recurred within one year was lower than that of children with Hp infection who did not recur, with statistically significant difference. While there was no statistically significant difference in Hb, SI and TIBC levels. It is suggested that children with low SF may have a high recurrence rate of Hp infection.

### Limitations

It includes a smaller sample size, the failure of excluding the impact of socio-economic factors on Hp infection, and the absence of correlation analysis of Hp infection with ID and IDA.

## CONCLUSION

In conclusion, low level of SF may be a risk factor for difficulty in eradication and recurrence after eradication in children with Hp infection. Meanwhile, low levels of Hb and SI are influential factors for difficulty in eradication in children with Hp infection.

### Authors’ Contributions:

**XL** and **YZ:** Carried out the studies, data collection, and drafted the manuscript, and are responsible and accountable for the accuracy or integrity of the work.

**QD** and **HD:** Performed the statistical analysis and participated in its design.

JB: Participated in acquisition, analysis, and interpretation of data and draft the manuscript.

All authors read and approved the final manuscript.

## References

[1] Gravina AG, Priadko K, Ciamarra P, Granata L, Facchiano A, Miranda A (2020). Extra-Gastric Manifestations of Helicobacter pylori Infection. J Clin Med.

[2] Khdair Ahmad F, Aladily TN, Altamimi M, Ajour M, Alsaber N, Rawashdeh M (2020). Helicobacter pylori Prevalence and Impact:A Histology-Based Report About Children from an Endemic Country. Int J Gen Med.

[3] George S, Lucero Y, Torres JP, Lagomarcino AJ, O'Ryan M (2020). Gastric Damage and Cancer-Associated Biomarkers in Helicobacter pylori-Infected Children. Front Microbiol.

[4] Okuda M, Lin Y, Kikuchi S (2019). Helicobacter pylori Infection in Children and Adolescents. Adv Exp Med Biol.

[5] Zhou Y, Ye Z, Huang J, Huang Y, Yan W, Zhang Y (2018). High prevalence and low spontaneous eradication rate of Helicobacter pylori infection among schoolchildren aged 7-12 years. Acta Paediatr.

[6] Ang TL, Ang D (2021). Helicobacter pylori Treatment Strategies in Singapore. Gut Liver.

[7] Savoldi A, Carrara E, Graham DY, Conti M, Tacconelli E (2018). Prevalence of Antibiotic Resistance in Helicobacter pylori:A Systematic Review and Meta-analysis in World Health Organization Regions. Gastroenterology.

[8] Al Kirdy F, Rajab M, El-Rifai N (2020). Helicobacter pylori Infection:Clinical, Endoscopic, and Histological Findings in Lebanese Pediatric Patients. Int J Pediatr.

[9] Santos MLC, De Brito BB, Da Silva FAF, Sampaio MM, Marques HS, Oliveira E, Silva N (2020). Helicobacter pylori infection:Beyond gastric manifestations. World J Gastroenterol.

[10] Zhang Y, Bi J, Wang M, Deng H, Yang W (2022). Correlation between helicobacter pylori infection and iron deficiency in children. Pak J Med Sci.

[11] Santambrogio E, Orsucci L (2019). Helicobacter pylori and hematological disorders. Minerva Gastroenterol Dietol.

[12] Gupta PM, Perrine CG, Mei Z, Scanlon KS, Gupta PM (2017). Iron, Anemia, and Iron Deficiency Anemia among Young Children in the United States Nutrients. 2016;8:330. Nutrients.

[13] Wang YK, Kuo FC, Liu CJ, Wu MC, Shih HY, Wang SS (2015). Diagnosis of Helicobacter pylori infection:Current options and developments. World J Gastroenterol.

[14] Who W (2001). Iron deficiency anaemia:assessment, prevention, and control. A guide for programme managers. Geneva Switzer Who.

[15] Zhang M (2015). High antibiotic resistance rate:A difficult issue for Helicobacter pylori eradication treatment. World J Gastroenterol.

[16] Hu Y, Wan JH, Li XY, Zhu Y, Graham DY, Lu NH (2017). Systematic review with meta-analysis:the global recurrence rate of Helicobacter pylori. Aliment Pharmacol Ther.

[17] Sivapalasingam S, Rajasingham A, Macy JT, Friedman CR, Hoekstra RM, Ayers T (2014). Recurrence of Helicobacter pylori infection in Bolivian children and adults after a population-based “screen and treat”strategy. Helicobacter.

[18] Zhang Y, Dong Q, Tian L, Zhang S, Zuo N, Zhang S (2020). Risk factors for recurrence of Helicobacter pylori infection after successful eradication in Chinese children:A prospective, nested case-control study. Helicobacter.

[19] Boyanova L, Hadzhiyski P (2020). Helicobacter pylori infection is associated with anemia, weight loss or both conditions among Bulgarian children. Acta Microbiol Immunol Hung.

[20] Haile K, Yemane T, Tesfaye G, Wolde D, Timerga A, Haile A (2021). Anemia and its association with Helicobacter pylori infection among adult dyspeptic patients attending Wachemo University Nigist Eleni Mohammad Memorial Referral Hospital, Southwest Ethiopia:A cross-sectional study. PLoS One.

[21] Hudak L, Jaraisy A, Haj S, Muhsen K (2017). An updated systematic review and meta-analysis on the association between Helicobacter pylori infection and iron deficiency anemia. Helicobacter.

[22] Miernyk K, Bruden D, Zanis C, McMahon B, Sacco F, Hennessy T (2013). The effect of Helicobacter pylori infection on iron stores and iron deficiency in urban Alaska Native adults. Helicobacter.

[23] Muhsen K, Cohen D (2008). Helicobacter pylori infection and iron stores:a systematic review and meta-analysis. Helicobacter.

[24] Zuo NY, Zhang YD, Dong QW, Bi J, Liu X (2021). Effect of Anti-Hp treatment on nutritional status of children with Helicobacter Pylori-Positive Gastritis and its clinical significance. Pak J Med Sci.

[25] Haghi-Ashtiani MT, Monajemzadeh M, Motamed F, Mahjoub F, Sharifan M, Shahsiah R (2008). Anemia in children with and without Helicobacter pylori infection. Arch Med Res.

[26] Cappellini MD, Musallam KM, Taher AT (2020). Iron deficiency anaemia revisited. J Intern Med.

[27] Zhan JY, Zheng SS, Dong WW, Shao J (2020). Zhonghua Er Ke Za Zhi.

